# Nasopharyngeal carcinoma risk prediction *via* salivary detection of host and Epstein-Barr virus genetic variants

**DOI:** 10.18632/oncotarget.11144

**Published:** 2016-08-09

**Authors:** Qian Cui, Fu-Tuo Feng, Miao Xu, Wen-Sheng Liu, You-Yuan Yao, Shang-Hang Xie, Xi-Zhao Li, Zu-Lu Ye, Qi-Sheng Feng, Li-Zhen Chen, Jin-Xin Bei, Lin Feng, Qi-Hong Huang, Wei-Hua Jia, Su-Mei Cao, Ellen T. Chang, Weimin Ye, Hans-Olov Adami, Yi-Xin Zeng

**Affiliations:** ^1^ Sun Yat-sen University Cancer Center, State Key Laboratory of Oncology in South China, Collaborative Innovation Center for Cancer Medicine, Guangzhou, P. R. China; ^2^ Department of Medical Epidemiology and Biostatistics, Karolinska Institutet, Stockholm, Sweden; ^3^ Department of Epidemiology, Harvard School of Public Health, Boston, MA, USA; ^4^ Division of Epidemiology, Department of Health Research and Policy, Stanford University School of Medicine, Stanford, CA, USA; ^5^ Sihui Cancer Institute, Sihui, Guangdong, P. R. China

**Keywords:** nasopharyngeal carcinoma, saliva, case-control, risk prediction

## Abstract

Genetic susceptibility and Epstein-Barr virus (EBV) infection are important etiological factors in nasopharyngeal carcinoma (NPC). In this study, in southern China, where NPC is endemic, a single nucleotide polymorphism (SNP) in the EBV-encoded *RPMS1* gene (locus 155391: G > A [G155391A]) and seven host SNPs (rs1412829, rs28421666, rs2860580, rs2894207, rs31489, rs6774494, and rs9510787) were confirmed to be significantly associated with NPC risk in 50 NPC cases versus 54 hospital-based controls with throat washing specimens and 1925 NPC cases versus 1947 hospital-based controls with buffy coat samples, respectively. We established a strategy to detect the NPC-associated EBV and host SNPs using saliva samples in a single test that is convenient, noninvasive, and cost-effective and displays good compliance. The potential utility of this strategy was tested by applying a risk prediction model integrating these EBV and host genetic variants to a population-based case-control study comprising 1026 incident NPC cases and 1148 controls. Receiver operating characteristic (ROC) curve analysis revealed an area under the curve of the NPC risk prediction model of 0.74 (95% CI: 0.71−0.76). Net reclassification improvement (NRI) analysis showed that inclusion of the EBV SNP significantly improved the discrimination ability of the model (NRI = 0.30, *P* < 0.001), suggesting the promising value of EBV characteristics for identifying high-risk NPC individuals in endemic areas. Taken together, we developed a promising NPC risk prediction model via noninvasive saliva sampling. This approach might serve as a convenient and effective method for screening the population with high-risk of NPC.

## INTRODUCTION

Nasopharyngeal carcinoma (NPC) is a malignancy associated with Epstein-Barr virus (EBV). It is quite rare in most parts of the world, with incidence rates well below 1 per 100,000 person-years, but it is rather prevalent in southern China, southeast Asia and northern Africa [1]. Remarkably, it has a geographic distribution and strikingly high incidence in the Guangdong Province, southern China, with an incidence rates above 20 per 100,000. Moreover, NPC incidence has remained high for at least the last 30 years in Sihui County, Guangdong Province [2–4]. For early-stage NPC, radiotherapy is often curative [5], whilst patients diagnosed at advanced stages have poorer outcomes [6–8]. Thus prevention and early detection are key to reducing NPC burden.

Risk prediction models aim to identify individuals at high risk for certain diseases, such as cancer. Risk prediction models of 13 cancer sites, not including NPC, were compiled by the U.S. National Cancer Institute [9]. Risk factors such as patient demographics, behavioral characteristics, medical history, and genetic variants may be useful in identifying high-risk individuals for earlier or more frequent screening and for preventative cancer counseling [10].

Several genome-wide association studies (GWASs) of NPC recently have been published [11–14]. The largest GWAS, based on subjects of southern Chinese descent, confirmed independent associations with three genetic variants in the human leukocyte antigen (HLA) region (rs2860580, rs2894207 and rs28421666 ), and also revealed three new susceptibility loci (rs1412829, rs6774494, and rs9510787) [13]. In a GWAS meta-analysis, we also identified an NPC risk locus in the CLPTM1L-TERT region on chromosome 5p15.33 (rs31489) [15]. EBV infection is an early, possibly initiating event in the development of NPC and is thought to play a critical role in transforming nasopharyngeal epithelial cells into invasive cancer cells [16, 17]. In endemic regions, high-risk EBV variants might exist that contribute to NPC risk.

In the current study, we aimed to construct a new risk prediction model of NPC based on both host and EBV genetic variants determined through saliva testing. Our objective was to develop a noninvasive and convenient method to identify individuals at high risk for NPC.

## RESULTS

### Association of a SNP in the EBV genome with NPC

Based on genotyping for *RPMS1* using nested PCR and Sanger sequencing methods in study population 1, we found that the frequencies of G155391A were significantly higher in the 50 matched samples from NPC patients (84% in tumor biopsy samples and 82% in tumor washing samples) than that in 54 healthy throat washing samples (39%) (Table 1). The sex and age-adjusted ORs for NPC associated with the A vs. G genotype was 8.41 (95% CI = 3.13–22.62, *P* = 2.42 × 10^−5^) based on tumor biopsy samples, and 6.34 (95% CI = 2.52–15.98, *P* = 9.01 × 10^−5^) based on throat washing samples. These results confirmed the strong association of G155391A in *RPMS1* of EBV with NPC risk in patients from Guangdong Province.

**Table 1:** Epstein-Barr virus *RPMS1* G155391A variant in matched TB and TW samples from NPC patients and healthy donors (study population 1)

Gene	Variant	NPC patients		Healthy donors
		TB (%)	TW (%)	TW (%)
*RPMS1*	A	42 (84%)	41 (82%)	21 (39%)*
	G	7 (14%)	9 (18%)	29 (54%)
	A/G	1 (2%)	0 (0%)	4 (7%)
	Total	50	50	54

Abbreviations: TB, tumor biopsy; TW, throat washing

*P value < 0.0001 for variant A vs. variant G in tumor biopsy or throat washing samples from NPC patients versus throat washing samples from healthy donors.

### Association of the 7 host SNPs with NPC

All of the 7 host SNPs identified from GWAS (rs2860580, rs2894207, rs28421666, rs1412829, rs31489, rs6774494, and rs9510787) were highly significantly associated with NPC in study population 2 (Table 2). The ORs and *P* values were as follows: rs2860580, OR = 1.68, *P* = 7.62 × 10^−25^; rs2894207, OR = 1.57, *P* = 2.54 × 10^−12^ ; rs28421666, OR = 1.64, *P* = 4.16 × 10^−11^; rs1412829, OR = 1.23, *P* = 6.85 × 10^−3^ ; rs31489, OR = 1.26, *P* = 3.70 × 10^−5^; rs6774494, OR = 1.14, *P* = 7.69 × 10^−3^; and rs9510787, OR = 1.17, *P* = 9.23 × 10^−4^.

**Table 2:** Associations of host SNPs previously reported in genome-wide association studies with NPCrisk as measured in buffy coat samples (study population 2) or saliva samples (study population 3)

SNP	Chr.	Locus	Allele^a^	Buffy coat samples of 1925 cases and 1947 controls		Saliva samples of 1026 NPC cases and 1148 controls	
				*P* value^b^	OR (95% CI)^c^	*P* value^b^	OR (95% CI)^c^
rs2860580	6	HLA-A	C/T	7.62 ×10^−25^	1.68 (1.52, 1.85)	3.26 × 10^−15^	1.71 (1.50,1.96)
rs2894207	6	HLA-B/C	T/C	2.54 × 10^−12^	1.57 (1.39, 1.79 )	8.59 × 10^−14^	1.88 (1.59,2.22)
rs28421666	6	HLA-DQ/DR	A/G	4.16 × 10^−11^	1.64 (1.41, 1.89 )	7.97 × 10^−3^	1.34 (1.08,1.67)
rs1412829	9	CDKN2A/2B	T/C	6.85 × 10^−3^	1.23 (1.06, 1.42)	3.75 × 10^−5^	1.52 (1.25,1.85)
rs31489	5	CLPTM1L-TERT	C/A	3.70 × 10^−5^	1.26 (1.13,1.41)	7.62 × 10^−3^	1.22 (1.05,1.41)
rs6774494	3	MDS1-EVI1	A/G	7.69 × 10^−3^	1.14 (1.03, 1.25)	3.63 × 10^−2^	1.15 (1.01,1.30)
rs9510787	13	TNFRSF19	G/A	9.23 × 10^−4^	1.17 (1.07, 1.28)	5.92 × 10^−2^	1.13 (1.00,1.27)

Abbreviations: OR, odds ratio; CI, confidence interval; Chr.: chromosome

a. Risk allele/reference allele.

b. *P* values for trend were derived from Cochran-Armitage trend tests.

c. Adjusted for age and sex.

### Saliva for simultaneous identification of host and EBV genetic variants

Paired saliva and buffy coat specimens of 103 controls from the study population 3 were used. The genotyping results for the seven host SNPs and one EBV variant in 103 buffy coat samples from study population 3 were compared with those from paired saliva samples. The seven host SNPs were successfully genotyped in all tested samples, and the concordance rates for the seven human SNPs in the paired saliva and buffy coat samples were all 100% (Table 3). The EBV variant was genotyped in 70.87% (73/103) of the saliva samples but only 9.71% (10/103) of the buffy coat samples (Table 3), suggesting that saliva is more enriched with EBV than buffy coats.

**Table 3:** Call rate and concordance rate of SNPgenotyping results using paired saliva and buffy coat samples (study population 3)

SNP	Call rate		Genotyped successfully in both saliva and buffy coat (*n*)	Concordance rate
	Saliva	Buffy coat		
rs1412829	100%	100%	103	100%
rs28421666	100%	100%	103	100%
rs2860580	100%	100%	103	100%
rs2894207	100%	100%	103	100%
rs31489	100%	100%	103	100%
rs6774494	100%	100%	103	100%
rs9510787	100%	100%	103	100%
EBV G155391A	71%	10%	8	75%

Abbreviations: EBV: Epstein-Barr virus

### Association of the host and EBV SNPs simultaneously identified in saliva with NPC risk

Based on simultaneous genotyping for host and EBV genetic variants in saliva samples from study population 3 (population-based case-control study), the three SNPs in the *HLA* region were significantly associated with NPC, i.e., rs2860580 (OR = 1.71, *P* = 3.26 × 10^−15^), rs2894207 (OR = 1.88, *P* = 8.59 × 10^−14^), and rs28421666 (OR = 1.34, *P* = 7.97 × 10^−3^) (Table 2), confirming GWAS results. Regarding the SNPs outside the *HLA* region, the previous associations of rs1412829 at *CDKN2A/2B* (OR = 1.52, *P* = 3.75 × 10^−5^), rs31489 at *CLPTM1L-TERT* (OR = 1.22, *P* = 7.62 × 10^−3^), and rs6774494 at *MDS1-EVI1* (OR = 1.15, *P* = 3.63 × 10^−2^) with NPC were replicated (Table 2). At the *TNFRSF19* locus, rs9510787 showed a borderline significant association with NPC (OR = 1.13, *P* = 5.92 × 10^−2^) consistent with previous findings [13].

G155391A was also significantly associated with NPC in study population 3 (OR = 5.74, 95% CI = 4.42–7.46, *P* = 2.56 × 10^−39^) (Table 4). Because NPC risk was also increased among those with the GA genotype, albeit nonsignificantly, we combined GA and G carriers for subsequent analyses.

**Table 4:** Association of variant G155391A in Epstein-Barr virus *RPMS1* with NPC risk in saliva samples from population-based NPC cases and controls (study population 3)

	Cases (%)	Controls (%)	*P* value*	OR (95% CI)*
G(reference)	92 (13%)	359 (45%)		
A	610 (85%)	416 (52%)	2.56 ×10^−39^	5.74 (4.42,7.46)
A/G	15 (2%)	31 (4%)	0.057	1.89 (0.98,3.66)

Abbreviations: OR: odds ratio; CI: confidence interval

*Adjusted for age and sex.

### Risk prediction models based on the host and EBV genetic variants

**Figure 1:**
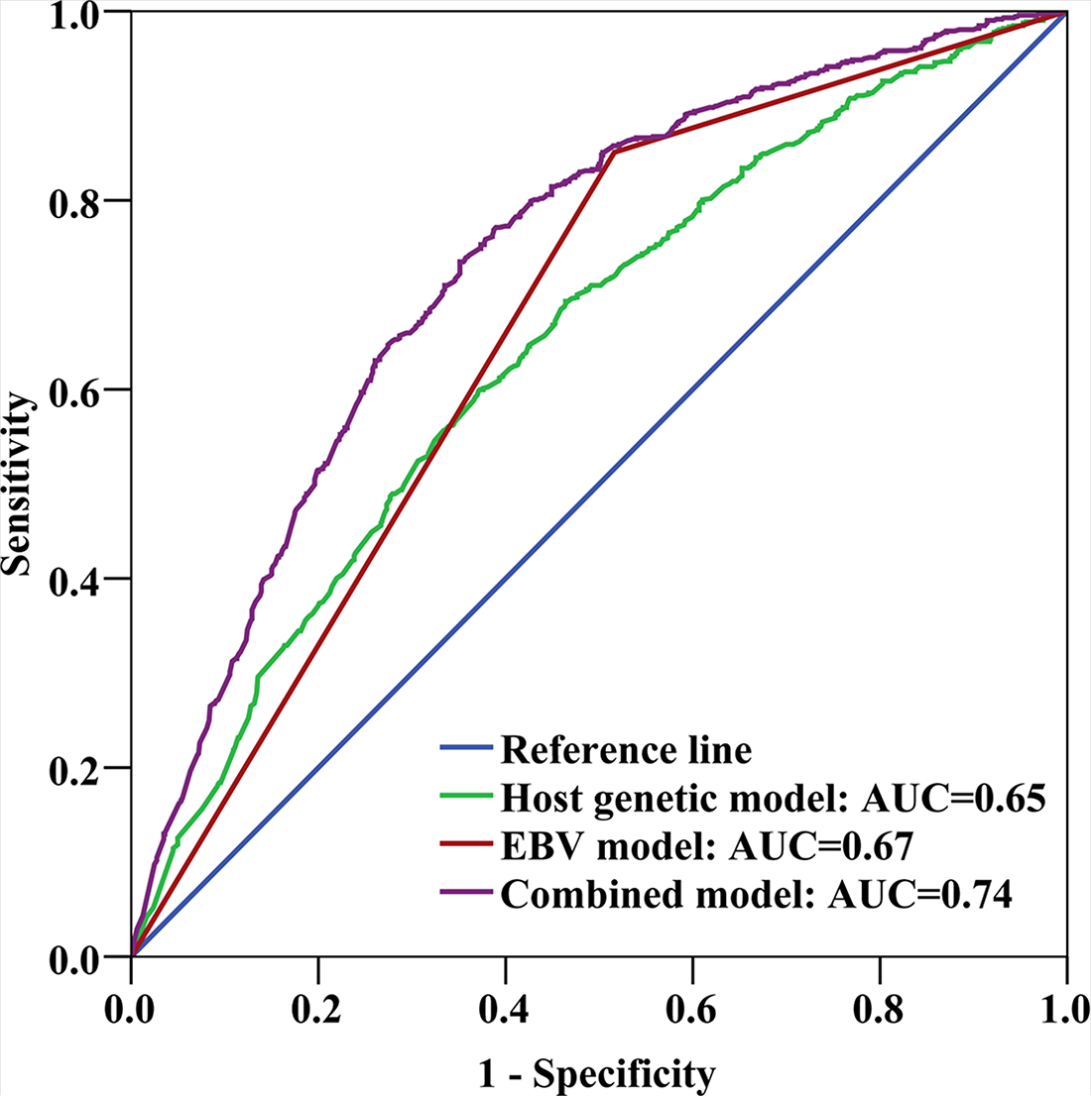
Receiver-operating characteristic (ROC) curve analysis Areas under the ROC curves (AUCs) were used as measures of the predictive power of the risk-assessment models based on host and EBV genetic variants. The host genetic model factors were 7 SNPs: rs1412829, rs28421666, rs2860580, rs2894207, rs31489, rs6774494, and rs9510787. The EBV model factor was G155391A. The combined model factors were the EBV variant and the 7 host SNPs.

ROC curve analysis revealed that the area under the curve (AUC) of the NPC risk prediction model that included the seven host SNPs and the EBV variant was 0.74 (95% CI = 0.71–0.76). This combined model substantively improved the risk prediction performance compared with the models including only the seven host SNPs (AUC = 0.65, 95% CI = 0.62−0.68) or only the EBV variant (AUC = 0.67, 95% CI = 0.64−0.69) (Figure 1, Supplementary Table 1). Adding age and sex (which were matched) did not change the AUC. We also performed the ROC curves in males and females, the AUC were 0.74 and 0.75, respectively (Supplementary Figures).

To determine whether the combined host and EBV model provides better classification results than the model based solely on host genetic markers, we calculated the NRI, setting the predicted risk threshold at 0.2 or 0.3 (Supplementary Table 2). Based on these calculations, the NRI was estimated to be 0.30 (*P* < 0.001), suggesting an improvement in prediction performance due to the inclusion of the EBV variant.

## DISCUSSION

We developed and tested a risk prediction model incorporating host and EBV genetic variants as a tool to identify individuals at high risk for NPC in an endemic population. The AUC of the full model was 0.74 in the population-based case-control study and addition of the EBV variant clearly improved the performance of the risk prediction model over the use of host genetic variants alone. Moreover, we established a strategy to simultaneously detect host and EBV genetic variants using a small amount of DNA in saliva. These results represent an advance in NPC-related genotyping methodology and in the prediction of NPC development using a practical approach.

Previous studies demonstrated associations of host SNPs and certain EBV variants with risk of NPC, using DNA from buffy coat and throat washing samples, respectively. However, such genotyping information is difficult to obtain because the procedures for separately collecting and genotyping the buffy coat and throat washing samples are complex. Saliva is an efficient, cost-effective, and noninvasive approach to examining predictors or biomarkers of complex diseases, and compliance is high because self-collection of saliva is feasible, painless and non-invasive, thus could improve the donor care [18]. In addition, previous findings show that saliva may be a suitable source of human DNA for SNP analysis [19]. In many studies, infectious disease markers detected in blood are also found in saliva. For instance, viral DNA/RNA, antibodies, and viral antigens have been detected in saliva, and their levels strongly correlate with those in blood samples [20, 21]. We speculated that saliva might be a good source of both human and EBV DNA [22, 23]. Indeed, our results showed high concordance rates of host and EBV genotypes between blood and saliva, and high call rates of both host and EBV genetic variants in saliva samples. Therefore, our findings demonstrate that saliva is an appropriate material for simultaneously identifying host and EBV genetic variants, and that saliva sampling might be convenient for large-scale population risk prediction of NPC.

Eight of 103 paired samples were successfully examined using both saliva and buffy coat samples, and 6 of the samples displayed no difference; thus, the genotyping results for saliva did not completely correspond to those for buffy coat. The number of EBV genomes was previously estimated as a median value of only 7 using 10^6^ B cells from the peripheral blood of healthy individuals [24]. Evidence also has indicated that the distribution and interchange of viral strains among peripheral blood mononuclear cells, plasma, and saliva are complex [25].

We confirmed the contributions of host genetic factors, particularly involving the *HLA* locus, to NPC etiology. Our independent analyses of the G155391A variant in EBV *RPMS1* in tumor biopsy and throat washing from study population 1 and in saliva from study population 3 also consistently revealed its strong association with NPC. Thus, G155391A in EBV *RPMS1* could serve as a valuable indicator for high risk of NPC in southern China. Furthermore, our results support that the EBV strain in tumor biopsy and throat washing may have the same origin. It has been demonstrated that EBV within intact oropharyngeal epithelium was derived from EBV-infected salivary cells through cell-to-cell contact [26]. Therefore, examining EBV strain in throat washing or saliva samples could replace the need for doing so in tumor biopsy.

Cancer risk prediction models may identify individuals at high risk who could benefit from targeted interventions. For instance, based on a risk prediction model [27, 28], an interactive risk assessment tool was designed to estimate a woman's risk of developing invasive breast cancer (http://www.cancer.gov/bcrisktool/) to help guide prevention strategies [29]. In addition, because human papillomaviruses 16 and 18 cause most cases of cervical cancer [30, 31], vaccines are now available to prevent initial infection with these strains. Multiple etiological factors are believed to be involved in NPC development, including genetic susceptibility, EBV infection, and diet [32, 33]. Thus, we could potentially utilize our risk prediction model in regions with high NPC incidence to identify high-risk groups for prevention or screening strategies specific to this population.

Although EBV antibody and DNA levels in serum are considered sufficiently sensitive and specific for NPC screening [34], the EBV antibody level depends on the host immune response to EBV infection and changes over time. The first NPC predictive genetic model did not include EBV antibody titers as predictors [35]. Another study showed that combining the most significant host SNPs with EBV IgA antibody status, which is presently used as a biomarker for NPC, did not improve the AUC estimate for NPC diagnosis [36]. Our results also showed that detection of circulating antibodies (VCA and EBNA1 IgA) can identify NPC patients with good performance and no significant differences in EBV antibody levels in subgroups of subjects defined by different EBV genetic variants (data not shown). EBV seromarkers might appear to be useful only for early detection of NPC, whereas other risk predictors may be useful for long-term NPC risk prediction. A more frequent antibody-based screening could be applied among individuals who screen positive by the long-term prediction of DNA-based tests.

More efforts are required to further improve our NPC risk prediction model. Adding informative factors such as family history might improve the predictive ability, and a long-term prospective cohort study is needed to validate any risk prediction model. Given recent developments in the sequencing of the EBV genome, a more valuable approach would be to derive whole genome sequences to identify the possibility of more significant EBV strain variation contributing to NPC risk. Hence, a large prospective study including environmental factors, host genetic factors, EBV characteristics (EBV antibodies, EBV DNA levels and EBV variations), family history and their interactions would be expected to lead to the construction of a more comprehensive risk prediction model.

In summary, we established a strategy to determine host and NPC-associated EBV genetic variants via noninvasive saliva sampling. We further developed a promising NPC risk prediction model integrating host and EBV genetic variants. This approach might provide a convenient and effective method to identify individuals who are at high risk for NPC development, thus reducing the NPC burden in endemic populations.

## MATERIALS AND METHODS

### Study population 1: EBV genotyping

Fifty-four histologically confirmed nasopharyngeal carcinoma (NPC) patients with individually matched samples from tumor biopsy (fresh or paraffin embedded) and throat washing were diagnosed at and enrolled from the Sun Yat-sen University Cancer Center in Guangzhou, China, between 2005 and 2007. The selection criteria for patients were Cantonese, not belonging to NPC family, without any close relative being NPC patient and newly diagnosed.

Sixty healthy controls with throat washing samples were enrolled from the physical examination center at the First Affiliated Hospital of Sun Yat-sen University. The selection criteria for control subjects included no individual history of cancer and matched to NPC cases by age, gender, residential region and ethnics.

### Study population 2: Host genotyping

Buffy coat samples were collected from 2023 histologically confirmed NPC cases treated at the Sun Yat-sen University Cancer Center between 2005 and 2010 and from 2009 healthy controls free of NPC, other cancers, or infectious diseases identified from the physical examination center at the First Affiliated Hospital of Sun Yat-sen University. All subjects were residing in the province of Guangdong. The characteristics of these subjects are summarized in Supplementary Table 3.

### Study population 3: Risk prediction model testing

Between 2010 and 2014 in Zhaoqing, Guangdong Province, 1026 population-based NPC cases were identified and enrolled through a rapid case ascertainment system involving a network of physicians in the area who diagnose or treat NPC. Through random selection from total population registries, 1148 population-based control subjects were identified and enrolled, with frequency matching to the cases by age, sex, and area of residence. Participation rates were approximately 81% among cases and 83% among controls.

Saliva samples were collected into vials containing an equal volume of prepared lysis buffer (50 mM Tris, pH 8.0, 50 mM EDTA, 50 mM sucrose, 100 mM NaCl, 1% SDS) as previously described [19]. Venous blood was collected simultaneously. Saliva specimens were provided by approximately 93% of participating cases and 89% of participating controls, while blood specimens were provided by approximately 98% and 83% of cases and controls, respectively. Each participant completed a detailed interview conducted face-to-face by a trained interviewer employing a structured questionnaire. The characteristics of these subjects are summarized in Supplementary Table 4.

### Ethics statement

Each subject provided informed consent, and the institutional review boards of all participating institutions approved this collaborative study.

### DNA extraction

DNA was isolated from tumor biopsy, throat washing, and buffy coat samples using a commercial DNA extraction kit (Qiagen, Germany). DNA from 200 μl of saliva or buffy coat samples in the population-based case-control study (study population 3) was automatically extracted using Chemagic STAR (Hamilton Robotics, Sweden) according to the manufacturer's instructions. DNA concentration greater than 10 ng/μl was required.

### Nested PCR and Sanger sequencing

To detect specific EBV genomic variants linked to NPC development, we previously sequenced several EBV-encoded genes, including *EBNA1*, *LMP1*, and the BamHI-A rightward transcripts (*BARTs*) family, in NPC cases and controls from Guangdong Province [37]. The most striking finding was a significant association between a single nucleotide polymorphism (SNP) in the EBV-encoded *RPMS1* gene (locus 155391: G > A, referred to here as G155391A, resulting in the alternation of Asp to Asn) and NPC risk. *RPMS1* encodes a major part of mRNA of the *BARTs* family and is regularly transcribed in NPC tissues [38]. Thus, G155391A in *RPMS1* of EBV may represent a specific EBV variant in the NPC-endemic region of southern China that could serve as an indicator of high risk of NPC in this population.

For the present study, we interrogated the trend of nucleotide polymorphisms in *RPMS1* were same in EBV DNA samples from tumor biopsy and throat washing. The *RPMS1* fragment was amplified by nested polymerase chain reaction (PCR) using two primer pairs (RPMS1-1/RPMS1-2 and RPMS1-3/RPMS1-4; Supplementary Table 5) and PCR Master Mix (Promega). DNA from the EBV-positive C666 cell line and the absence of DNA were used as the positive and negative controls, respectively. The sequences of the PCR products were identified by Sanger sequencing.

### Genotyping

Seven host SNPs previously identified in GWASs (rs2860580, rs2894207, rs28421666, rs9510787, rs6774494, rs1412829, and rs31489), together with 264 405 autosomal SNPs mostly enriched in the exonic regions, were genotyped in buffy coat samples from study population 2 using customized Illumina Infinium HumanExome BeadChips. For quality control of the exome-wide data, the SNP filtering criteria included remaining singleton autosomal SNPs, minor allele frequency above 1%, genotyping call rate above 98%, and no deviation from Hardy-Weinberg equilibrium (*P* ≥ 0.05 in controls); the sample filtering criteria included call rate above 98% for SNPs per sample. After further examination for relatedness and population structure, 1925 cases and 1947 controls were retained for association analysis of the seven GWAS-identified SNPs.

Simultaneous genotyping for the seven human SNPs and the single EBV variant was performed in saliva samples from study population 3 using the Agena Bioscience MassArray platform. The primers used are shown in Supplementary Table 6. Eighty-two samples were re-genotyped due to a bad spectrum or no allele information. After quality validation, based on call rate (100%) and no deviation from Hardy-Weinberg equilibrium (*P* ≥ 0.05 in controls), 1026 cases and 1148 controls were included for further analysis.

### Statistical analysis

We used logistic regression analysis to estimate odds ratios (ORs) and 95% confidence intervals (CIs) for associations with NPC risk adjusting for age and sex, assuming an additive model for host genetic variants. For comparisons across SNPs, we used the ORs for the high-risk alleles rather than minor alleles. *P* values for trend were derived from Cochran-Armitage trend tests. All reported *P* values are two-sided.

Using a logistic regression method, we constructed risk prediction models based on the seven host genetic variants, the single EBV variant, or all eight variants in saliva samples from study population 3 in which the EBV variant was successfully detected. Receiver operating characteristic (ROC) curve analysis was used to evaluate the performance of the model.

To quantify discriminatory improvement for the model including the EBV variant and the seven host variants compared with the model including only the host variants, we computed the net reclassification improvement (NRI). The predicted risk threshold was set at 0.2 or 0.3, and we used a reclassification table to evaluate how accurately the two models assigned the individuals to the low, intermediate, and high-risk categories.

The statistical analyses were performed using SPSS (version 16.0) and R (version 2.14.0).

## SUPPLEMENTARY MATERIALS FIGURES AND TABLES


