# Supplementary material for: Nasopharyngeal carcinoma risk prediction via salivary detection of host and Epstein-Barr virus genetic variants
Source: Oncotarget. 2016 Aug 9;8(56):95066–74. doi: 10.18632/oncotarget.11144 (PMC5707005; doi:10.18632/oncotarget.11144)
Supplement: Supplementary file 1 [file oncotarget-08-95066-s001.pdf]

Nasopharyngeal carcinoma risk prediction *via* salivary detection of host and Epstein-Barr virus genetic variants

Supplementary Materials

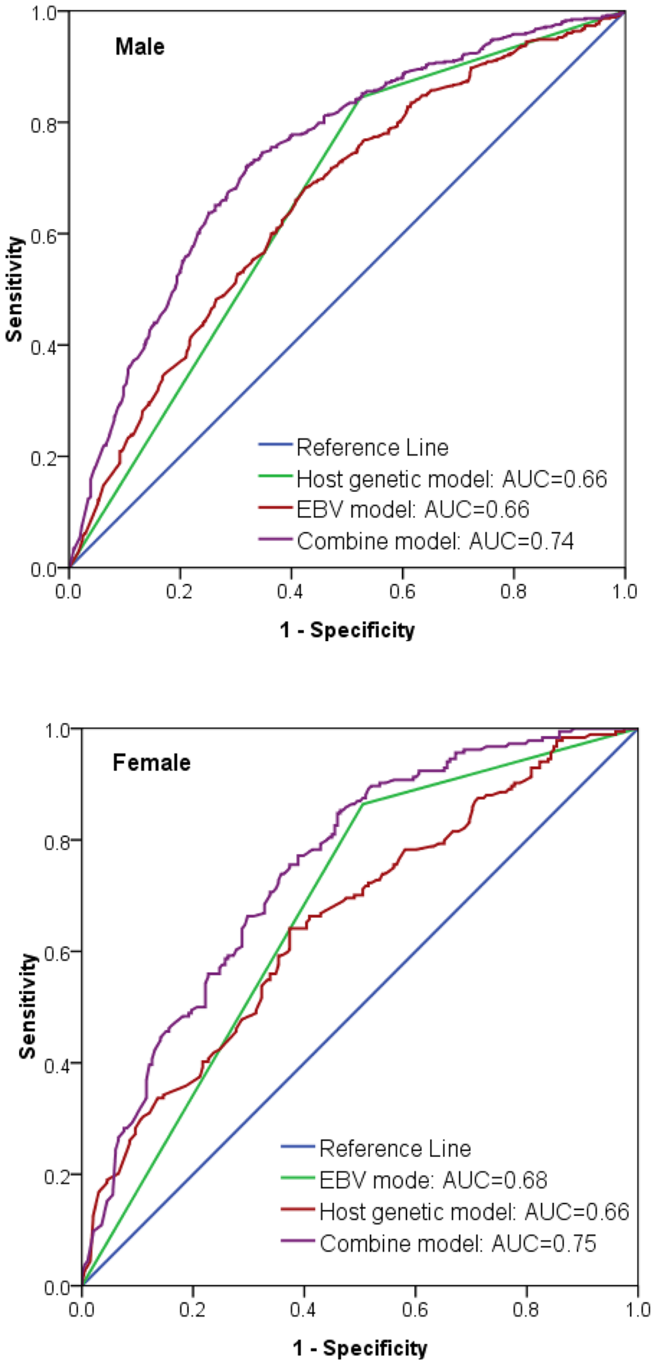

Supplementary Figures: Receiver-operating characteristic (ROC) curve analysis in males and females. Areas under the ROC curves (AUCs) were used as measures of the predictive power of the risk-assessment models based on host and EBV genetic variants. The host genetic model factors were 7 SNPs: rs1412829, rs28421666, rs2860580, rs2894207, rs31489, rs6774494, and rs9510787. The EBV model factor was G155391A. The combined model factors were the EBV variant and the 7 host SNPs.

Supplementary Table S1: AUC of the genetic only, EBV only and combine models

	Model	AUC (95%CI)	*P* value[e]
Genetic model[a]	$logitP_1^d = 0.31 \times rs1412829 + 0.29 \times rs28421666 + 0.49 \times rs2860580 + 0.58 \times rs2894207 + 0.13 \times rs31489 + 0.11 \times rs6774494 + 0.19 \times rs9510787 - 3.36$	0.65 (0.62–0.68)	< 0.001
EBV model[b]	$logitP_2^d = 1.65 \times$ EBV G155391A − 1.29	0.67 (0.64–0.69)	< 0.001
Combine model[c]	$logitP_3^d = 0.27 \times rs1412829 + 0.24 \times rs28421666 + 0.48 \times rs2860580 + 0.45 \times rs2894207 + 0.06 \times rs31489 + 0.13 \times rs6774494 + 0.17 \times rs9510787 + 1.57 \times$ EBV G155391A − 3.97	0.74 (0.71–0.76)	< 0.001

a. Genetic model factors including rs1412829, rs28421666, rs2860580, rs2894207, rs31489, rs6774494, rs9510787.
b. EBV model factor including EBV variation based on the Loc155391.
c. Combine model factors including host genetic and EBV variants.
d. P was predicted probability of NPC occurrence.
e. *P* value was estimated using the Omnibus Test for the models.

Supplementary Table S2: Reclassification of nasopharyngeal carcinoma (NPC) cases and population-based controls based on the combined (host and Epstein-Barr virus) risk prediction model versus the host genetic risk prediction model

Host genetic risk model	Combined risk model				Reclassified		
	[0, 0.2]	[0.2, 0.3]	[0.3, 1]	Total	Increased risk	Decreased risk	Net correctly reclassified
Controls							
[0, 0.2]	14	7	0	21	41 (5%)	347 (43%)	−0.38
[0.2, 0.3]	60	2	34	96			
[0.3, 1]	101	186	402	689			
Total	175	195	436	806			
NPC cases							
[0, 0.2]	3	4	0	7	31 (4%)	89 (12%)	−0.08
[0.2, 0.3]	8	0	27	35			
[0.3, 1]	25	56	594	675			
Total	36	60	621	717			
NRI = 0.30 (95% CI: 0.26–0.34), *P* < 0.001							

NRI: net reclassification improvement, CI: confidence interval.

Supplementary Table S3: Age and gender distributions of subjects in study population 2 (2,023 nasopharyngeal carcinoma cases and 2,009 healthy controls)

	Cases	Controls
Male	1,526	1,354
Female	497	655
Mean age (years, SD)	46 (11.5)	45 (11.9)
Total number	2,023	2,009

SD: standard deviation.

Supplementary Table S4: Age and gender distributions of subjects in study population 3 (1,026 nasopharyngeal carcinoma cases and 1,148 population-based controls)

Variables	Cases	Controls
Sex (N, %)		
Male	743 (72%)	839 (73%)
Female	283 (28%)	309 (27%)
Age (years)		
Mean (SD)	47.9 (11.0)	49.1 (10.9)
< 30	150 (5%)	44 (4%)
30–40	202 (20%)	194 (17%)
41–50	374 (36%)	419 (37%)
51–60	266 (26%)	304 (26%)
> 60	134 (13%)	187 (16%)
Total number	1,026	1,148

SD: standard deviation.

Supplementary Table S5: Two sets of nested primers for amplification of the Epstein-Barr virus *RPMS1* gene

Outer	RPMS1-1	gct ggg ttga tgc tgt aga tg
Outer	RPMS1-2	agg gtc tgg acg tgg agt ttg
Inner	RPMS1-3	aga agg cgt aga gca tgt cca g
Inner	RPMS1-4	gag tac gac tgt gag gtg ggc g

Supplementary Table S6: Primers used for the Sequenom MassArray single nucleotide polymorphism (SNP) genotyping of 7 host SNPs and 1 Epstein-Barr virus variant

Locus ID	2nd PCRP	1st PCRP	UEP_SEQ	EXT1_SEQ	EXT2_SEQ
rs1412829	ACGTTGGATGCATGCTTTGGGAAACTCTAC	ACGTTGGATGCCATTGCTATGGTTACTATC	CTACCCATGAGATTCATATTCAAGC	CTACCCATGAGATTCATATTCAAGCA	CTACCCATGAGATTCATATTCAAGCG
rs28421666	ACGTTGGATGCTGTTGGGAGGAGAAAAATG	ACGTTGGATGATCTATACTGTGATATTTAT	CATTAAGATCCTTCCTGTTTT	CATTAAGATCCTTCCTGTTTTC	CATTAAGATCCTTCCTGTTTTT
rs2860580	ACGTTGGATGTTCCCTGCTTCATTGTGCAT	ACGTTGGATGTTACAGTCATGGCAGAAGTG	CTCTCTCCTGTCACCAC	CTCTCTCCTGTCACCACA	CTCTCTCCTGTCACCACG
rs2894207	ACGTTGGATGGCTTATGTTTTCTTCTAAGAG	ACGTTGGATGTGCAAAAGAATAAAGCTGG	TTTTCTTCTAAGAGTTCTCTAAT	TTTTCTTCTAAGAGTTCTCTAATA	TTTTCTTCTAAGAGTTCTCTAATG
rs31489	ACGTTGGATGTCGCATTCCACCTGTTTACG	ACGTTGGATGTACACTTTCAGCCTGGTGAC	ctTTCTTCTTCCTCTTTAAAAGT	ctTTCTTCTTCCTCTTTAAAAGTC	ctTTCTTCTTCCTCTTTAAAAGTA
rs6774494	ACGTTGGATGTACGGTAGATGCCATTAAGG	ACGTTGGATGCTATCTTACTTACATTTACC	gcGGAAAACAGTCAATATGTCAC	gcGGAAACAGTCAATATGTCACC	gcGGAAAACAGTCAATATGTCACT
rs9510787	ACGTTGGATGGGCTGACCTGCAACTCTTAG	ACGTTGGATGGATTTATTACTTATTGGTGC	gTCATAGTCTTAGAAGACAGC	gTCATAGTCTTAGAAGACAGCA	gTCATAGTCTTAGAAGACAGCG
EBV_Loc155391	ACGTTGGATGAGGCCCTGGCCTTCGATAT	ACGTTGGATGGATATCTGCAGGATCAGGTC	CCTTCGATATCGAGTGT	CCTTCGATATCGAGTGTC	CCTTCGATATCGAGTGTT
